# Monocyte-derived Langerhans cells express Delta-like 4 induced by peptidoglycan and interleukin-4 mediated suppression

**DOI:** 10.3389/fimmu.2025.1532620

**Published:** 2025-02-13

**Authors:** Rei Ono, Kohei Maeda, Toshihiro Tanioka, Takeo Isozaki

**Affiliations:** ^1^ Department of Pathogenesis and Translational Medicine, Showa University Graduate School of Pharmacy, Tokyo, Japan; ^2^ Department of Rheumatology, Showa University Graduate School of Medicine, Tokyo, Japan

**Keywords:** Langerhans cells, monocyte, autoimmune diseases, Delta-like 1, Delta-like 4, T cell, psoriasis, toll-like receptor

## Abstract

T cells contribute to immunotherapy and autoimmune pathogenesis and Langerhans cells (LCs) have a substantial ability to activate T cells. *In vitro*-generated monocyte-derived LCs (Mo-LCs) are useful models to study LC function in autoimmune diseases and to test future LC-based immunotherapies. Although dendritic cells (DCs) expressing high levels of Delta-like 4 (DLL4^+^ DCs), which is a member of the Notch ligand family, have greater ability than DLL4^−^ DCs to activate T cells, the induction method of human DLL4^+^ DCs has yet to be determined. The aim of this study is to establish whether Mo-LCs express DLL4 and establish the induction method of antigen presenting cells, which most potently activate T cells, similar to our previously established induction method of human Mo-LCs. We compared the ratios of DLL4 expression and T cell activation via flow cytometry among monocyte-derived cells, which have a greater ability than the resident cells to activate T cells. Here, we discovered that Mo-LCs expressed DLL4, which most potently activated T cells among monocyte-derived cells, and that Mo-LCs and DLL4 expression were induced by DLL4, granulocyte macrophage colony-stimulating factor, and transforming growth factor-β1. Additionally, peptidoglycan was required for DLL4 expression, whereas interleukin-4 repressed it. These findings provide insights into the roles of DLL4-expressing cells such as DLL4^+^ Mo-LCs in human diseases, which will assist with the development of more effective therapeutic strategies in the future.

## 1 Introduction

Psoriasis, an autoinflammatory immune disease, is driven by infections stimulating dendritic cells (DCs) to release IL-23; this activates T cells, resulting in the production of IL-17, which amplifies psoriatic inflammation (1). Langerhans cells (LCs), a type of DC expressing langerin, have a substantial ability to activate T cells (2, 3). Human LCs have been reported to be involved in psoriatic lesions (4). Monocyte-derived LCs (Mo-LCs), induced during inflammation (5), rather than resident LCs, are required for psoriasis-like changes in mouse skin (6, 7). However, since profound anatomical and immunological differences are obvious when comparing mice and humans, it is challenging to establish a method for generating human Mo-LCs *in vitro* to probe the role of Mo-LCs in psoriasis pathogenesis (4, 8, 9).

Notch signaling, which is initiated through receptor (in mammalians, Notch 1, 2, 3, and 4)-ligand (Delta-like 1 [DLL1], DLL3, DLL4, Jagged1 [JAG1] and JAG2) interactions, plays a crucial role in cell fate decisions and differentiation processes (10, 11). Previous studies imply a required mechanical function that is met by the immobilization of the Notch ligand either in a membrane or on a plastic surface (12, 13). Hoshino et al. reported that DLL1, in concert with granulocyte macrophage colony-stimulating factor (GM-CSF) and transforming growth factor (TGF)-β1, induces the differentiation of human CD14^+^ blood monocytes into Mo-LCs (14), which is considered as the most reproducible and simple culture condition leading Mo-LCs to display bona fide LC features (13–16). However, the specialized materials required to immobilize DLL-1 are difficult to obtain, which may explain the small number of subsequent reports on DLL-1-induced Mo-LCs (17–20). Using DLL1 and DLL4 expressed on mouse OP9 cells, Milne et al. reported that DLL4 generates more Mo-LCs than DLL1 (21); however, this method mixes proteins derived from OP9 cells in terms of biochemical analysis. We previously established a simple, reproducible, and efficient immobilizing method using only commercial Notch ligands and culture plates (13). We sought to elucidate the optimal Notch ligand for generating Mo-LCs using the immobilizing method, as previous works have not compared the Mo-LC induction ratios among all Notch ligands (14, 21, 22).

Aside from their contribution to psoriasis, T cells also contribute to immunotherapy and autoimmune pathogenesis (23, 24). Monocytes can differentiate into DCs, LCs, and macrophages (Mφs) (5, 25), which have a greater ability than the resident cells to activate T cells (6, 26, 27). However, no previous study has compared the T cell activation ratios among these monocyte-derived cells, which is addressed in the current study.

Activation of toll-like receptor (TLR) signaling induces high levels of DLL4 in CD1c^+^ DCs and plasmacytoid DCs, and DCs expressing high levels of DLL4 (DLL4^+^ DCs) have greater ability than DLL4^−^ DCs to activate T cells (24). Additionally, emerging data suggest a relationship between DLL4 and psoriasis (28, 29). Thus, we considered it likely that DLL4 is expressed on monocyte-derived cells, which potently activate T cells and induce inflammatory conditions, including psoriasis.

We show here that DLL4 most potently induces Mo-LCs among Notch ligands, and that Mo-LCs express DLL4 by stimulation with the TLR2 ligand peptidoglycan (PGN), which is suppressed by interleukin (IL)-4. Additionally, DLL4^+^ Mo-LCs most potently activate T cells among monocyte-derived cells.

## 2 Materials and methods

### 2.1 Reagents

The reagents used in this study included recombinant human (rh) DLL1 (BioLegend, San Diego, CA), rhDLL4, rhGM-CSF, rhTGF-β1, rhIL-4 (Peprotech, Cranbury, NJ), rhDLL3, rhJAG1 (AdipoGen Life Sciences, San Diego, CA), rhJAG2 (Cloud Clone Corp, Houston, TX), PGN, Polyinosinic-polycytidylic acid (Poly(I:C)), lipopolysaccharide (LPS) from *Escherichia coli* (L4516), and imiquimod (IMQ) (Sigma-Aldrich, St. Louis, MO).

### 2.2 Immobilizing the Notch ligand

Briefly, 3 μg/ml Notch ligand dissolved in phosphate-buffered saline (PBS) was added to uncoated 24-well plates with hydrophobic surfaces that are normally used for suspension cell cultures (MS-8024R; SUMILON, Tokyo, Japan). The plates were centrifuged at 750 rpm for 7 min at room temperature, before removing the supernatant and adding 6 μg/ml Notch ligand dissolved in PBS. The plates were then centrifuged at 750 rpm for 7 min at room temperature and washed with PBS.

### 2.3 Cell preparation

Samples from 7 healthy volunteers were obtained after they signed an informed consent approved by Showa University Research Ethics Review Board (Tokyo, Japan) (Approval number: 22-250-B).

Peripheral blood mononuclear cells were isolated from heparinized blood via Ficoll-Paque PLUS (Cytiva, Uppsala, Sweden) density gradient (1.077) centrifugation in Leucosep (Greiner Bio-One, Kremsmünster, Austria). After depleting platelets, CD14^+^ monocytes were isolated followed by CD4^+^ T cells using CD14 and CD4 antibody-conjugated magnetic microbeads (Miltenyi Biotec, Bergisch Gladbach, Germany), respectively, according to the manufacturer’s instructions. The isolated CD14^+^ monocytes (1 × 10^6^/ml) were cultured in Roswell Park Memorial Institute (RPMI) 1640 medium (FujiFilm Wako Pure Chemical Corporation, Osaka, Japan) supplemented with 10% heat-inactivated fetal bovine serum (FBS) (Biosera, Nuaille, France) and penicillin (100 U/ml)/streptomycin (100 μg/ml) (FujiFilm Wako Pure Chemical Corporation, Osaka, Japan). The Mo-LCs were generated from CD14^+^ monocytes in culture with GM-CSF and TGF-β1 on a Notch ligand-immobilized plate, the Mo-DCs were generated from CD14^+^ monocytes in culture with GM-CSF and IL-4, and the Mφs were generated from CD14^+^ monocytes in culture with GM-CSF. The concentrations of GM-CSF, IL-4, and TGF-β1 were 10 ng/ml.

### 2.4 Fluorescence-activated cell sorting (FACS)

On day 7, the cells were adjusted to a concentration of 3 × 10^6^ cells/ml and incubated at 4°C for 40 min with the appropriate antibodies. After washing with ice-cold PBS containing 0.2% bovine serum albumin (FujiFilm Wako Pure Chemical Corporation, Osaka, Japan) and 2 mM ethylenediaminetetraacetic acid (FujiFilm Wako Pure Chemical Corporation, Osaka, Japan), the cells were analyzed on a FACSAria II using the FACSDiva software (Becton-Dickinson and Company, Franklin Lakes, NJ). The phycoerythrin-labeled monoclonal antibody (mAb) to DLL4, and langerin (CD207); allophycocyanin-labeled mAb to langerin (CD207); and Pacific blue-labeled mAb to human leukocyte antigen-DR (BioLegend, San Diego, CA) were used for flow cytometry.

### 2.5 Activation of monocyte-derived cells by TLR ligands

On day 6, the cells were cultured in the presence or absence of 5 μg/ml of the TLR2 ligand PGN, 25 μg/ml of the TLR3 ligand Poly(I:C), 1 μg/ml of the TLR4 ligand LPS, or 1 μg/ml of the TLR7/8 ligand IMQ for 24 h.

### 2.6 CellTrace Violet labeling

CD4^+^ T cells (1 × 10^6^/mL) were incubated for 20 min at 37°C with 1 μl CellTrace Violet (Thermo Fisher Scientific, Waltham, MA). Next, 4 ml RPMI 1640 supplemented with 10% FBS and penicillin (100 U/ml)/streptomycin (100 μg/ml) was added to the cells and incubated for 5 min. Subsequently, the cells were centrifuged and resuspended in pre-warmed Iscove’s Modified Dulbecco’s Medium (Thermo Fisher Scientific, Waltham, MA) supplemented with 10% CTS immune cell serum replacement (Thermo Fisher Scientific, Waltham, MA) and penicillin (100 U/ml)/streptomycin (100 μg/ml), before coculturing with monocyte-derived cells at ratios of 2.5:1, 5:1, and 1:1.5 for 5 days. CellTrace Violet-labeled cells were analyzed for fluorescence intensity using a FACSAria II flow cytometer.

### 2.7 Statistical analysis

Statistical significance between different experimental groups was analyzed using Tukey’s test to determine the best inducer of Mo-LCs or DLL4 expression or the best activator of T cells. Statistical analyses were performed using the JMP Pro 15 software for Windows (SAS Institute Inc., Cary, NC). A probability (p) value <0.05 was considered statistically significant.

## 3 Results

### 3.1 DLL4 most potently induces Mo-LCs

**Figure 1:**
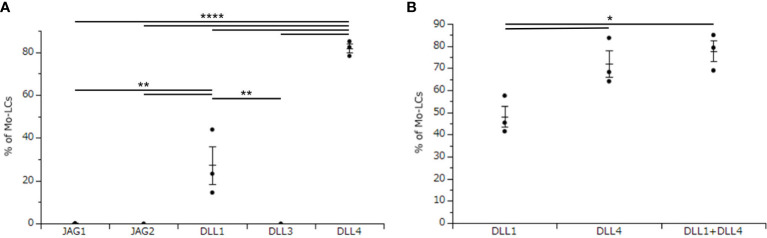
Delta-like 4 (DLL4) most potently induces monocyte-derived Langerhans cells (Mo-LCs). **(A)** Percentage of Mo-LCs (langerin^+^ cells) induced on all Notch ligand-immobilized plates. **(B)** Percentage of Mo-LCs induced on DLL1-, DLL4-, and DLL1+DLL4-immobilized plates. **(A, B)** The Mo-LCs were generated from CD14^+^ monocytes in culture with granulocyte macrophage colony-stimulating factor (GM-CSF) and transforming growth factor (TGF)-β1 on the Notch ligand-immobilized plates. After 7 days of culture, the cells were analyzed by Fluorescence-activated cell sorting (FACS). In this assay, we used blood samples from **(A)** donor No.1-3, **(B)** donor No.4-6. *p < 0.05, **p < 0.01, ****p < 0.0001.

The aim of this study is to establish the induction method of antigen presenting cells, which most potently activate T cells. To use Mo-LCs for this study, we first sought to establish which Notch ligands can give rise to Mo-LCs *in vitro*. To this end, CD14^+^ monocytes were cultured with GM-CSF and TGF-β1 on a Notch ligand-immobilized plate for 7 days, before analyzing the induction of Mo-LCs by FACS. We found that both DLL1 and DLL4 induced large amounts of Mo-LCs, but DLL4 most efficiently and reproducibly induced Mo-LCs (**Figure 1A**).

DLL1 is the Notch ligand detected in the epidermis (30), and a previous study demonstrated increased expression of DLL4 in psoriasis lesional skin compared to unaffected skin (28, 29). Thus, both DLL1 and DLL4 are likely to be detected in psoriasis lesional skin. Hence, we next sought to determine whether DLL1 and DLL4 synergize to induce Mo-LCs. Our results showed that although DLL4 significantly induced Mo-LCs, it showed no synergistic effect when used in combination with DLL1 (**Figure 1B**).

### 3.2 Mo-LCs express DLL4 by TLR ligand stimulation

**Figure 2:**
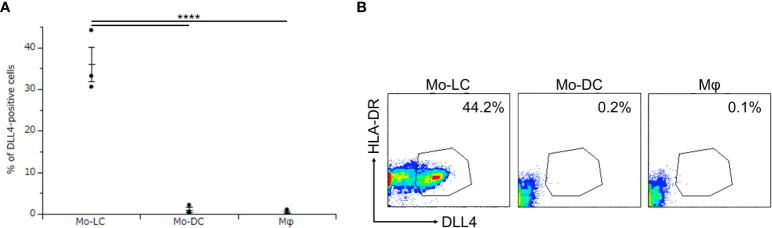
Mo-LCs express DLL4 by toll-like receptor (TLR) ligand stimulation. **(A, B)** Percentage of DLL4-positive cells (DLL4^+^ human leukocyte antigen (HLA)-DR^+^ cells) within Mo-LC, monocyte-derived dendritic cell (Mo-DC), and macrophage (Mφ). The Mo-LCs were generated from CD14^+^ monocytes in culture with GM-CSF and TGF-β1 on DLL4-immobilized plates, the Mo-DCs were generated from CD14^+^ monocytes in culture with GM-CSF and interleukin (IL)-4, and the Mφs were generated from CD14^+^ monocytes in culture with GM-CSF. The DLL4 expression on Mo-LC, Mo-DC, and Mφ after activation by Polyinosinic-polycytidylic acid (Poly(I:C)) and lipopolysaccharide (LPS) for 24 h was determined by FACS on day 7. **(A)** In this assay, we used blood samples from donor No.1-3. ****p < 0.0001. **(B)** Two-color flow cytometry of the cultured cells was performed to measure DLL4 and HLA-DR.

Activation of TLR signaling induces high levels of DLL4 in CD1c^+^ DCs and plasmacytoid DCs, and DLL4^+^ DCs have greater ability than DLL4^−^ DCs to activate T cells (24). We considered it likely that DLL4 is expressed also on monocyte-derived cells, which potently activate T cells and induce inflammatory conditions, including psoriasis (6, 24, 26–29). We have previously reported that Mo-LCs produce significant amounts of cytokines related to the pathogenesis of psoriasis compared to Mo-DCs and Mφs in response to Poly(I:C) or LPS (13). Thus, we next sought to establish whether monocyte-derived cells expressed DLL4 in response to Poly(I:C) and LPS. The Mo-LCs were generated from CD14^+^ monocytes in culture with GM-CSF and TGF-β1 on DLL4-immobilized plate, the Mo-DCs were generated from CD14^+^ monocytes in culture with GM-CSF and IL-4, and the Mφs were generated from CD14^+^ monocytes in culture with GM-CSF. Our results showed that Mo-LCs expressed high amounts of DLL4, whereas Mo-DCs and Mφs did not (**Figures 2A, B**).

### 3.3 PGN most potently induces DLL4 expression on Mo-LCs

**Figure 3:**
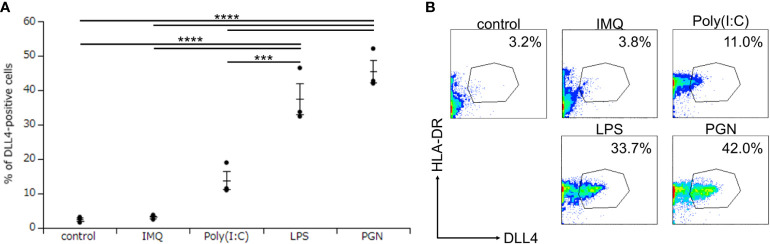
Peptidoglycan (PGN) most potently induces DLL4 expression on Mo-LCs. **(A, B)** Percentage of DLL4-positive cells induced by control (without TLR ligands), PGN, Poly(I:C), LPS, and imiquimod (IMQ). The Mo-LCs were generated from CD14^+^ monocytes in culture with GM-CSF and TGF-β1 on DLL4-immobilized plates. The DLL4 expression on Mo-LC after activation by TLR ligands for 24 h was determined by FACS on day 7. **(A)** In this assay, we used blood samples from donor No.1-3. ***p < 0.001, ****p < 0.0001. **(B)** Two-color flow cytometry of the cultured cells was performed to measure DLL4 and HLA-DR.

Although we discovered that Mo-LCs express DLL4 (**Figures 2A, B**), it remains unclear at which differentiation stages, including common myeloid progenitor, Mφ and DC progenitor, DC precursors and immature DCs, the endpoint of hematopoietic stem cells/hematopoietic progenitor cells to become DLL4^+^ DC precursor cells is established and why Mo-DCs cannot become DLL4^+^ DCs (24). To answer these questions, we investigated the critical regulators of DLL4 expression using DLL4^+^ Mo-LCs. We first investigated which TLR ligands can upregulate DLL4 expression. We analyzed the DLL4 expression on Mo-LCs after activation by PGN, Poly(I:C), LPS, and IMQ for 24 h. We found that PGN most efficiently and reproducibly induced DLL4 expression (**Figures 3A, B**).

### 3.4 DLL4 most potently induces DLL4 expression on Mo-LCs

**Figure 4:**
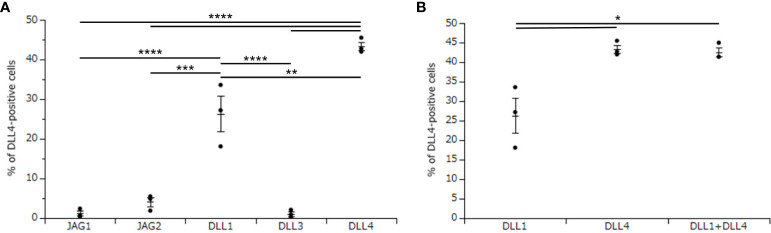
DLL4 most potently induces DLL4 expression on Mo-LCs. **(A)** Percentage of DLL4-positive cells induced on all Notch ligand-immobilized plates. **(B)** Percentage of the DLL4-positive cells induced on DLL1, DLL4, and DLL1+DLL4-immobilized plates. **(A, B)** The Mo-LCs were generated from CD14^+^ monocytes in culture with GM-CSF and TGF-β1 on the Notch ligand-immobilized plates. The DLL4 expression on Mo-LC after activation by PGN for 24 h was determined by FACS on day 7. In this assay, we used blood samples from donor No.1, 2, and 4. *p < 0.05, **p < 0.01, ***p < 0.001, ****p < 0.0001.

To investigate the critical regulators of DLL4 expression using DLL4^+^ Mo-LCs induced by PGN (**Figures 3A, B**), next, we investigated which Notch ligands can upregulate the expression of DLL4. Similar to the induction of Mo-LCs (**Figure 1**), DLL1 and DLL4 induced high levels of DLL4 expression, and DLL4 most efficiently and reproducibly induced DLL4 expression (**Figure 4A**).

We next investigated whether DLL1 and DLL4 synergize in the induction of DLL4 expression. Our results showed no synergistic effect when using DLL4 together with DLL1 (**Figure 4B**).

### 3.5 DLL4 and GM-CSF are required to induce DLL4 expression, which is promoted and repressed by TGF-β1 and IL-4, respectively

**Figure 5:**
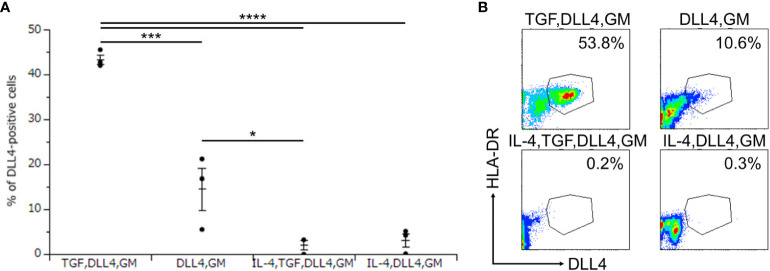
DLL4 and GM-CSF are required for the induction of DLL4 expression, which is promoted and repressed by TGF-β1 and IL-4, respectively. **(A, B)** Percentage of DLL4-positive cells induced by immobilized DLL4, GM-CSF, TGF-β1, and IL-4. The CD14^+^ monocytes were cultured in the presence or absence of immobilized DLL4, GM-CSF, TGF-β1, and IL-4. The DLL4 expression on monocytes after activation by PGN for 24 h was determined by FACS on day 7. **(A)** In this assay, we used blood samples from donor No.1, 2, and 4. *p < 0.05, ***p < 0.001, ****p < 0.0001. **(B)** Two-color flow cytometry of the cultured cells was performed to measure DLL4 and HLA-DR.

To investigate the critical regulators of DLL4 expression using DLL4^+^ Mo-LCs induced by PGN (**Figures 3A, B**) and DLL4 (**Figures 4A, B**), next, we searched for cytokines that induce DLL4 expression given that the results shown in **Figure 2** suggest that GM-CSF, TGF-β1, DLL4, and IL-4 regulate DLL4 expression on monocyte-derived cells. We compared the DLL4 expression ratios in combination with these cytokines. As a result, we found that DLL4 and GM-CSF were required for the induction of DLL4 expression, which was promoted and repressed by TGF-β1 and IL-4, respectively (**Figures 5A, B**). DLL4, DLL4+TGF-β1, and DLL4+TGF-β1+IL-4 induced very few cells, while GM-CSF, TGF-β1, IL-4, DLL4+IL-4, GM-CSF+TGF-β1, GM-CSF+IL-4, TGF-β1+IL-4, GM-CSF+TGF-β1+IL-4, and the control (without cytokines) did not induce DLL4^+^ cells (data not shown).

### 3.6 DLL4^+^ Mo-LCs most potently activate CD4^+^ T cells

**Figure 6:**
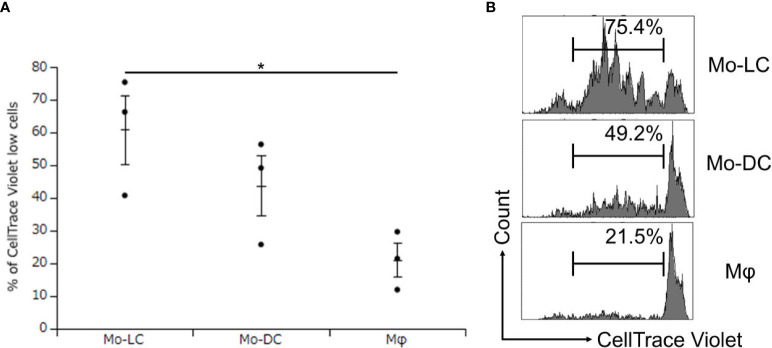
DLL4^+^ Mo-LCs most potently activate CD4^+^ T cells. **(A, B)** Percentage of CellTrace Violet low cells activated by DLL4^+^ Mo-LC, Mo-DC, and Mφ. The DLL4^+^ Mo-LCs were generated from CD14^+^ monocytes in culture with GM-CSF and TGF-β1 on DLL4-immobilized plates, the Mo-DCs were generated from CD14^+^ monocytes in culture with GM-CSF and IL-4, and the Mφs were generated from CD14^+^ monocytes in culture with GM-CSF. After activation by PGN for 24 h, the monocyte-derived cells were cocultured with CellTrace Violet-labeled CD4^+^ T cells from the same donor on day 7. After 5 days, the CellTrace Violet dilution was analyzed by FACS. **(A)** In this assay, we used blood samples from donor No.5-7. *p < 0.05.

No previous study has compared the T cell activation ratios between Mo-DCs, Mo-LCs, and Mφs. Finally, DLL4^+^ Mo-LCs induced by immobilized DLL4 (**Figures 4A, B**), GM-CSF, TGF-β1 (**Figures 5A, B**), and PGN (**Figures 3A, B**) were compared to Mo-DCs and Mφs stimulated by PGN for their ability to activate T cells. The Mo-DCs were generated from CD14^+^ monocytes in culture with GM-CSF and IL-4, and the Mφs were generated from CD14^+^ monocytes in culture with GM-CSF. The results suggested that DLL4^+^ Mo-LCs were most effective at activating T cells (**Figures 6A, B**).

**Figure 7:**
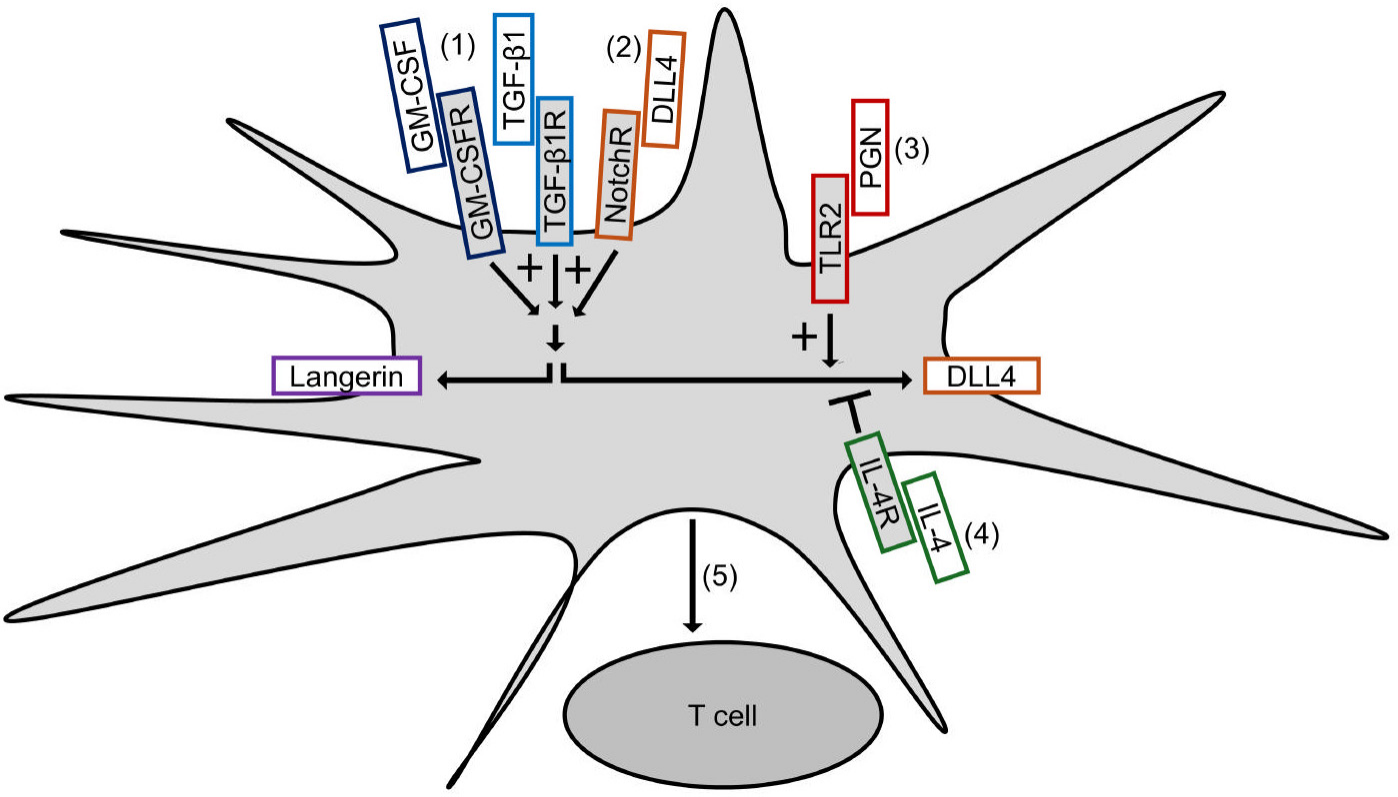
Induction of DLL4^+^ Mo-LCs activating T cells most potently. (1) GM-CSF and TGF-β1 not only induce Mo-LCs but also DLL4 expression. (2) DLL4 most potently induces Mo-LCs and DLL4 expression among Notch ligands. (3) PGN most potently induces DLL4 expression among TLR ligands. (4) IL-4 represses DLL4 expression. (5) DLL4^+^ Mo-LCs most potently activate T cells among monocyte-derived cells.

**Figure 7** summarizes our results and depicts our proposed mechanism of DLL4 expression on Mo-LCs. The aim of this study was to establish whether Mo-LCs express DLL4 and establish the induction method of antigen presenting cells, which most potently activate T cells. As a result, Mo-LCs expressed DLL4. DLL4^+^ Mo-LCs most potently activated CD4^+^ T cells among the monocyte-derived cells, and DLL4, GM-CSF, TGF-β1, and PGN are the factors that most efficiently induce DLL4^+^ Mo-LCs. Additionally, the factors responsible for inducing Mo-LCs are almost the same factors responsible for inducing DLL4 expression.

## 4 Discussion

In this study, we sought to identify the Notch ligands necessary for monocytes to give rise to Langerhans-like cells. We proved that DLL1 and DLL4 induced Mo-LCs, and that DLL4 most efficiently and reproducibly induced Mo-LCs (**Figure 1**). These results are in contrast to those reported by Schwentner et al. (22), although this is different from previous reports (14, 21), possibly due to different protocols used for the *in vitro* differentiation and Schwentner et al. observed high donor variability in terms of fold induction of Mo-LCs. In terms of the mechanism by which DLL4 induces Mo-LCs, activation of Notch signaling and the concomitant loss of Kruppel-like factor 4 may be involved (19).

We discovered that Mo-LCs expressed DLL4, and Mo-LCs and DLL4 expression were induced by comparable factors (**Figure 7**). We discovered that DLL1 or DLL4 was required for the induction of DLL4 expression, and that IL-4 repressed it, suggesting that Mo-DCs cannot become DLL4^+^ DCs (24) because of the presence of IL-4 and the absence of DLL1 or DLL4 in the Mo-DC generation process.

DLL4 induced Mo-LCs and DLL4 expression more than DLL1 (**Figures 1**, **4**). As DLL4 exhibits a binding affinity for Notch receptors that is stronger than DLL1 (31) or DLL1 and DLL4 can activate distinct targets (32), differences in induction ratios seem likely. It is speculated that DLL4^+^ Mo-LCs play important roles in psoriatic lesions given the increased expression of DLL4 in psoriasis lesional skin (28, 29), the relationship between Mo-LC and psoriasis (5–7, 13), the high level of expression of TLR2 on blood monocytes in patients with psoriasis (33), the fact that the TLR2 ligand PGN most potently induces DLL4 expression (**Figure 3**), and that DLL4^+^ Mo-LCs most potently activate T cells (**Figure 6**). Furthermore, the induction of Mo-LCs by DLL4 (**Figure 1**) and DLL4 expression on Mo-LCs (**Figure 4**) might provoke a positive feedback loop of DLL4^+^ Mo-LC generation (9, 34, 35) that is necessary for the persistence of the disease. However, DLL1 may be the first Notch ligand to induce Mo-LCs or DLL4 expression given that DLL1 is detected in unaffected skin (30) and DLL4 expression increases in psoriasis lesional skin (28, 29).

TLR-mediated nuclear factor kappa B (NF-κB) signaling stimulates the production of IL-6, which binds to the IL-6 receptor and activates STAT3. STAT3 induces DLL1 expression that activates Notch signaling and boosts NF-κB-induced IL-6, which transduces the stabilization of STAT3 activation (34). Such a mechanism may be involved in DLL4 expression because both NF-κB and STAT3 are required for the induction of DLL4^+^ DCs (24) and DLL4 expression on a stromal cell line is augmented by IL-6 via STAT3 activation (36). Moreover, not only IL-6 but also IL-23 might be involved because PGN and LPS induce IL-23 (8, 13) and IL-23 induces STAT3 (8, 37, 38); alternatively, DLL4 might directly induce STAT3 (39). TLR-induced JAG1 expression is strongly dependent on the Notch master transcriptional regulator RBP-J, as well as on upstream components of the Notch pathway γ-secretase and Notch1 and Notch2 receptors (9). However, among Notch ligands, only DLL1 and DLL4 induce DLL4 expression (**Figure 4**), suggesting that TLR-induced DLL4 expression is not dependent on the Notch pathway but on the DLL1 and DLL4 pathway. TGF-β1 might contribute to DLL4 expression by promoting NF-κB (40, 41) or STAT3 through Smad3 (42–44) or cooperatively with Notch signaling (45). IL-4 might suppress DLL4 expression by suppressing TGF-β1 (46), NF-κB (47, 48), or yes-associated protein (YAP) (49) because YAP promotes DLL4 (50) and NF-κB (51) and DLL4 promotes YAP (52). Th17 differentiation has been shown to be efficiently elicited by not Mo-DCs but cDCs activated by PGN (8). Additionally, among TLR ligands, PGN most efficiently induced DLL4 expression on Mo-LCs but not on Mo-DCs (**Figures 2**, **3**). These results suggest that DLL4 expression in response to PGN stimulation has the potential to induce Th17 differentiation. Both PGN and LPS induced high levels of DLL4 expression on the surface of Mo-LCs, whereas Poly(I:C) and IMQ did not (**Figure 3**). One explanation for this may be that bacteria induce DLL4 expression on Mo-LCs more than viruses, or because Mo-LCs express TLRs 2 and 4 more than TLRs 3, 7, and 8 (13). However, these mechanisms are still speculative, and further research is needed to determine the signaling pathways.

DLL4 has been reported to increase LPS-stimulated cytokines by enhancing NF-κB activation (53–55). Contrary to these findings, other studies have proposed that TLR-mediated proinflammatory cytokines are reduced upon overexpression of NICD in mouse peritoneal Mφs (56). Our results relating to DLL4 expression suggest that different TLR ligands, Notch ligands, or cytokines can evoke such contradictions.

A fundamental difference between the human and murine systems has been highlighted (4, 8, 9). However, compared to mouse LCs, our knowledge of human LCs remains very limited, and with the discovery of human LC subsets, it is crucial to explore their features and functions under both physiological and pathological conditions from the perspective of subsets (57). DLL4^+^ Mo-LCs (**Figure 2**), the discovery of a novel human LC subset, may contribute to the exploration.

Because mechanistic studies that induce DLL4 in DCs are important for better defining the ontogeny of DLL4^+^ DCs and targeting these cells for immunotherapy (24), the discovery of the induction method of DLL4-expressing cells (**Figure 7**) may contribute to devising a novel strategy using the cells or signaling pathways through such as DLL4, TGF-β1, IL-4, or langerin for human patients with autoinflammatory immune diseases such as psoriasis (24, 35, 58, 59). In addition, *in vitro*-generated Mo-LCs with GM-CSF, TGF-β1, and DLL4 closely resemble their *in vivo* skin counterparts and are not only a useful, easily accessible tool to study different functions of LCs but may also help to elucidate their potential for immunotherapies (27). With the discovery that DLL4^+^ Mo-LCs most potently activated T cells among the monocyte-derived cells (**Figure 6**), targeting DLL4^+^ Mo-LCs may offer a promising therapeutic strategy such as in cancer treatment (24, 27, 35, 57, 60).

While targeting DLL4-expressing cells holds promise for the treatment of various human diseases, the limitations of the study are the potential off-target effects (35, 61). Emerging research areas and potential new therapeutic strategies, including a nanocarrier (35) and a synthetic Notch (62), are being explored to increase the specificity of targeting. The discovery that DLL4 expression is dependent not on the Notch pathway but on the DLL1 and DLL4 pathways (**Figure 4**) may contribute to improving therapeutic efficacy. The development of more selective inhibitors targeting specific Notch ligands or receptors, or modulating specific downstream pathways, may help to overcome off-target effects (63). Moreover, understanding how DLL4 interacts with signaling molecules or pathways, including langerin, IL-4, and TGF-β1, might lead to combined therapeutic strategies that target multiple pathways for enhanced efficacy (35). Additionally, it is necessary to obtain a thorough understanding of the context-dependent roles of DLL4^+^ Mo-LCs (35, 64). Nevertheless, it will be intriguing to elucidate the roles of DLL4^+^ Mo-LCs because DLL4^+^ Mo-LCs may be responsible for T cell activation in autoinflammatory immune diseases such as psoriasis (5–7, 13, 24, 28, 29, 33, 35, 59) and may also be efficacious for cancer treatment (24, 27, 35, 57, 60). Stimulated and matured Mo-DCs and Mo-LCs can activate T cells more effectively (27, 65). Thus, we speculate that non-stimulated Mo-LCs would not activate T cells as robustly as PGN-treated Mo-LCs (**Figure 6**). Further studies should include comparisons with other stimulations, such as LPS or the standard DC-cytokine maturation cocktail containing IL-1β, IL-6, TNF-α, and prostaglandin E2 (65), to confirm whether Mo-LCs are better activators of T cell responses. Verification of the results using measures such as the division or proliferation index would also be valuable. Although Mo-DCs and Mφs were obtained using standard induction methods (60, 66), verifying their markers in future experiments would strengthen the conclusions. Finally, while we discovered that DLL4^+^ Mo-LCs most potently activate T cells among the monocyte-derived cells (**Figures 6**, **7**), further research is needed to explore whether langerin, DLL4, or another molecule derived from DLL4, TGF-β1, and GM-CSF is crucial for T cell activation.

In conclusion, we show here that Mo-LCs express DLL4 on the cell surface when activated by PGN, which is suppressed by IL-4. These findings provide insights into the roles of DLL4-expressing cells such as DLL4^+^ Mo-LCs in human diseases and the development of more effective therapeutic strategies.

## Data Availability

The original contributions presented in the study are included in the article/**Supplementary Material**. Further inquiries can be directed to the corresponding author.
