# Supplementary material for: Monocyte-derived Langerhans cells express Delta-like 4 induced by peptidoglycan and interleukin-4 mediated suppression
Source: Front Immunol. 2025 Feb 13;16:1532620. doi: 10.3389/fimmu.2025.1532620 (PMC11865044; doi:10.3389/fimmu.2025.1532620)
Supplement: Supplementary file 1 [file Supplementaryfile1.pdf]

frontiers

Supplementary Material

1 Supplementary Figures

1.1 Supplementary Figure 1

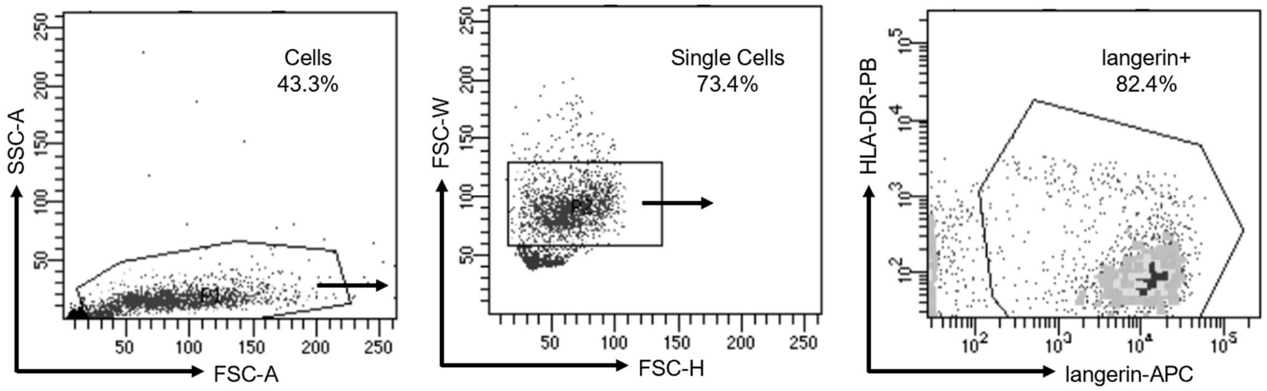

Supplementary Figure 1. Gating strategy for Mo-LCs. Dot plots representative of one donor illustrating the gating strategy for Mo-LCs. Mo-LCs were defined as Langerin^{+}.

1.2 Supplementary Figure 2

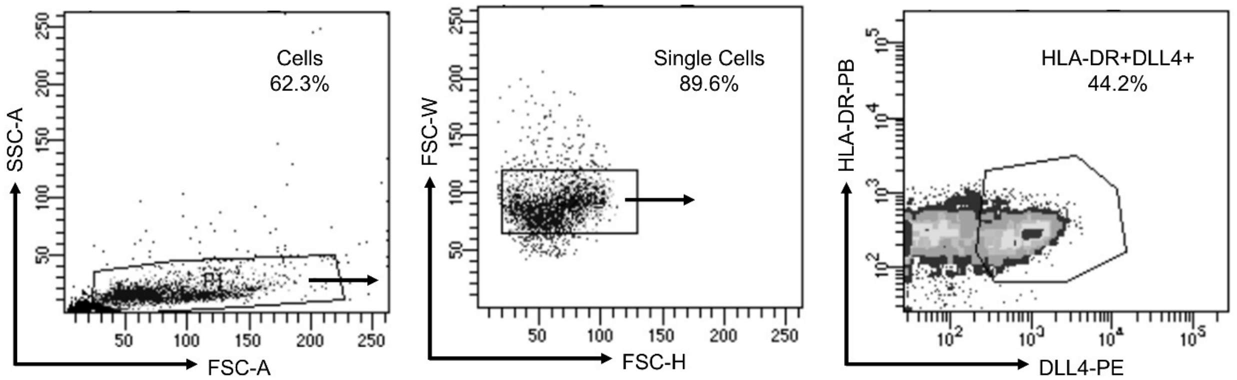

Supplementary Figure 2. Gating strategy for DLL4-positive cells. Dot plots representative of one donor illustrating the gating strategy for DLL4-positive cells. DLL4-positive cells were defined as HLA-DR^{+}DLL4^{+}.

1.3 Supplementary Figure 3

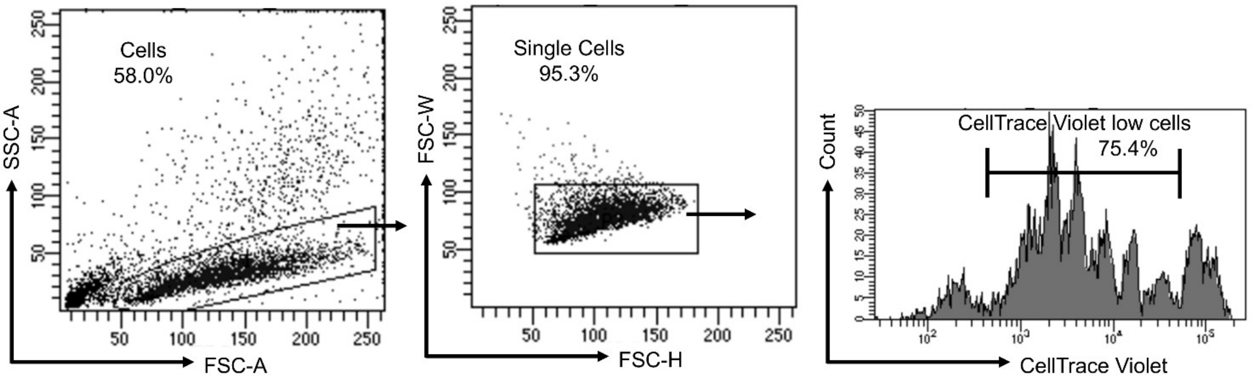

Supplementary Figure 3. Gating strategy for CellTrace Violet low cells. Dot plots representative of one donor illustrating the gating strategy for CellTrace Violet low cells.

1.4 Supplementary Figure 4

Figure 1	Figure 2	Figure 3	Figure 4	Figure 5	Figure 6
Immobilizing the Notch ligand<br>↓<br>PBMC were isolated<br>↓<br>Monocytes were isolated<br>↓<br>Mo-LCs were generated from monocytes in culture with GM-CSF and TGF-β1 on a Notch ligand-immobilized plate<br>↓<br>After 7 days of culture, % of Mo-LCs were analyzed by FACS	Immobilizing DLL4<br>↓<br>PBMC were isolated<br>↓<br>Monocytes were isolated<br>↓<br>Mo-LCs were generated from monocytes in culture with GM-CSF and TGF-β1 on DLL4-immobilized plate<br>Mo-DCs were generated from monocytes in culture with GM-CSF and IL-4<br>Mφs were generated from monocytes in culture with GM-CSF<br>↓<br>Activation by Poly(I:C) and LPS for 24 h<br>↓<br>After 7 days of culture, % of DLL4^{+} cells were analyzed by FACS	Immobilizing DLL4<br>↓<br>PBMC were isolated<br>↓<br>Monocytes were isolated<br>↓<br>Mo-LCs were generated from monocytes in culture with GM-CSF and TGF-β1 on DLL4-immobilized plate<br>↓<br>Activation by TLR ligands for 24 h<br>↓<br>After 7 days of culture, % of DLL4^{+} cells were analyzed by FACS	Immobilizing the Notch ligand<br>↓<br>PBMC were isolated<br>↓<br>Monocytes were isolated<br>↓<br>Mo-LCs were generated from monocytes in culture with GM-CSF and TGF-β1 on a Notch ligand-immobilized plate<br>↓<br>Activation by PGN for 24 h<br>↓<br>After 7 days of culture, % of DLL4^{+} cells were analyzed by FACS	Immobilizing DLL4<br>↓<br>PBMC were isolated<br>↓<br>Monocytes were isolated<br>↓<br>Monocytes were cultured in the presence or absence of immobilized DLL4, GM-CSF, TGF-β1, and IL-4<br>↓<br>Activation by PGN for 24 h<br>↓<br>After 7 days of culture, % of DLL4^{+} cells were analyzed by FACS	Immobilizing DLL4<br>↓<br>PBMC were isolated<br>↓<br>Monocytes and T cells were isolated<br>↓<br>Mo-LCs were generated from monocytes in culture with GM-CSF and TGF-β1 on DLL4-immobilized plate<br>Mo-DCs were generated from monocytes in culture with GM-CSF and IL-4<br>Mφs were generated from monocytes in culture with GM-CSF<br>↓<br>Activation by PGN for 24 h<br>↓<br>Mo-LCs, Mo-DCs, and Mφs were cocultured with CellTrace Violet-labeled T cells on day 7<br>↓<br>After 5 days, the CellTrace Violet dilution was analyzed by FACS

Supplementary Figure 4. A schematic timeline / experimental design for each figure.
